# Form Factors as Potential Imaging Biomarkers to Differentiate Benign vs. Malignant Lung Lesions on CT Scans

**DOI:** 10.3390/s22135044

**Published:** 2022-07-04

**Authors:** Francesco Bianconi, Isabella Palumbo, Mario Luca Fravolini, Maria Rondini, Matteo Minestrini, Giulia Pascoletti, Susanna Nuvoli, Angela Spanu, Michele Scialpi, Cynthia Aristei, Barbara Palumbo

**Affiliations:** 1 Department of Engineering, Università degli Studi di Perugia, Via Goffredo Duranti 93, 06125 Perugia, Italy; mario.fravolini@unipg.it; 2 Section of Radiation Oncology, Department of Medicine and Surgery, Università degli Studi di Perugia, Piazza Lucio Severi 1, 06132 Perugia, Italy; isabella.palumbo@unipg.it (I.P.); cynthia.aristei@unipg.it (C.A.); 3 Unit of Nuclear Medicine, Department of Medical, Surgical and Experimental Sciences, Università degli Studi di Sassari, Viale San Pietro 8, 07100 Sassari, Italy; maria.rondini01@ateneopv.it (M.R.); snuvoli@uniss.it (S.N.); aspanu@uniss.it (A.S.); 4 Section of Nuclear Medicine and Health Physics, Department of Medicine and Surgery, Università degli Studi di Perugia, Piazza Lucio Severi 1, 06132 Perugia, Italy; matteo.minestrini@ospedale.perugia.it (M.M.); barbara.palumbo@unipg.it (B.P.); 5 Department of Mechanical and Aerospace Engineering, Politecnico di Torino, Corso Duca Degli Abruzzi 24, 10129 Torino, Italy; giulia.pascoletti@polito.it; 6 Division of Diagnostic Imaging, Department of Medicine and Surgery, Piazza Lucio Severi 1, 06132 Perugia, Italy; michele.scialpi@unipg.it

**Keywords:** lung cancer, radiomics, form factors, computed tomography

## Abstract

Indeterminate lung nodules detected on CT scans are common findings in clinical practice. Their correct assessment is critical, as early diagnosis of malignancy is crucial to maximise the treatment outcome. In this work, we evaluated the role of form factors as imaging biomarkers to differentiate benign vs. malignant lung lesions on CT scans. We tested a total of three conventional imaging features, six form factors, and two shape features for significant differences between benign and malignant lung lesions on CT scans. The study population consisted of 192 lung nodules from two independent datasets, containing 109 (38 benign, 71 malignant) and 83 (42 benign, 41 malignant) lung lesions, respectively. The standard of reference was either histological evaluation or stability on radiological followup. The statistical significance was determined via the Mann–Whitney U nonparametric test, and the ability of the form factors to discriminate a benign vs. a malignant lesion was assessed through multivariate prediction models based on Support Vector Machines. The univariate analysis returned four form factors (Angelidakis compactness and flatness, Kong flatness, and maximum projection sphericity) that were significantly different between the benign and malignant group in both datasets. In particular, we found that the benign lesions were on average flatter than the malignant ones; conversely, the malignant ones were on average more compact (isotropic) than the benign ones. The multivariate prediction models showed that adding form factors to conventional imaging features improved the prediction accuracy by up to 14.5 pp. We conclude that form factors evaluated on lung nodules on CT scans can improve the differential diagnosis between benign and malignant lesions.

## 1. Introduction

According to the World Health Organisation, lung cancer is the second most common form of neoplastic disorder and the first cause of cancer-related deaths worldwide [1]. The most common forms of lung cancer are Non-Small Cell Lung Cancer (NSCLC), which accounts for ≈84% of the cases, followed by Small Cell Lung Cancer (SCLC) with ≈13% [2]. The American Cancer Society’s projections for 2022 place the number of new cases and fatalities in the USA at ≈230.000 and ≈130.000, respectively, with an overall chance of developing lung cancer in a lifetime of about 1 in 15 for men and 1 in 17 for women [2]. In Italy there were, in 2020, ≈41.000 newly diagnosed cases (≈67% men) and ≈13.300 deaths (≈69% men [3]).

The survival of patients with lung cancer strongly depends on the stage at which the disease is first detected, and early diagnosis is a key to maximise the treatment outcome. In Italy the 5-year overall survival is estimated at 16% for men and 23% for women [3]. At an early, stage lung cancer usually appears as a round, solid, subsolid, or ground-glass opacity (lung nodule), although only a small fraction of such abnormalities (estimated between 3.7% and 5.5%) actually represent malignancies [4]. The evaluation of suspicious lung lesions involves the assessment of clinical (e.g., age, sex, history of smoking, exposure, and other risk factors) and radiographic features such as size, margins, contour, density, and internal characteristics [5,6]. The updated guidelines (2017) from the Fleischner Society [7] recommend no routine followup for low-risk patients with solid and subsolid lung nodules smaller than 6 mm; in the other cases, the management may involve periodic CT scans, PET/CT imaging, and/or tissue sampling.

In recent years, quantitative computerised analysis of imaging data (*radiomics*) has introduced opportunities for the management of patients with suspicious and/or confirmed lung cancer [8,9,10,11,12,13]. The overall objective of radiomics is to extract quantitative data from the input images, which should be ideally repeatable, interpretable, and, of course, correlated with the clinical endpoint of interest [14]. The rationale behind this paradigm is that medical images are a source of diagnostic and prognostic data not directly revealed through traditional qualitative visual inspection [10,15,16,17,18,19,20]. The extraction of quantitative information in a mineable way also enables the resulting data to be fed into artificial intelligence algorithms to build automatic classification and/or regression models [21,22]. Furthermore, whereas other diagnostic procedures, such as biopsy, usually focus on a limited portion of the lesion, radiomics enables full-field analysis of the region of interest [23].

The radiomics pipeline involves six steps [12,21]: acquisition, preprocessing, segmentation, feature extraction, postprocessing, and data analysis. Feature extraction, in particular, consists of computing a set of quantitative parameters (*features*) from the imaging data. Ideally, the features should correlate with the clinical endpoint investigated—benignity or malignancy—in this case. Feature extraction methods can be classified into two main families: the ‘conventional’ (also referred to as ‘traditional’ or ‘hand-designed’) ones and those based on deep learning [24,25]. The conventional features can be further categorised into shape and texture features [15,26].

The literature has consistently reported the potential benefits of radiomics in several decision-making scenarios related to the management of patients with lung cancer. These include, among others, the discrimination between histological subtypes and between primary vs. metastatic lesions [27,28,29,30,31], prediction of the overall survival, disease-free survival, and response to therapy [32,33,34,35,36], and the detection of gene mutation status [37,38]. In particular, the role of deep learning and/or conventional features to help discriminate a benign vs. a malignant lung lesion on a CT scan has been assessed in a number of previous studies [39,40,41,42,43,44,45,46,47,48,49,50,51,52,53,54]. In this context, however, shape features have received little attention on their own, since they are usually investigated along with texture features to build high-dimensional predictive models in which the role of each single feature is not the main focus of the study [46,48,51,55]. Yet, shape features have a number of potential advantages, which make them particularly appealing in radiomics, such as intuitive interpretation and robustness to changes in the acquisition and reconstruction settings [56,57]. Among the three-dimensional shape features most commonly used to discriminate benign vs. malignant lung lesions on CT scans are the compactness, sphericity, spiculation, spherical disproportion, and the surface-to-volume ratio [48,55,58,59,60].

The objective of this study was to investigate one specific class of shape features, *form factors*, and, in particular, the potential of *elongation*, *flatness*, and *compactness* as recently defined in [61] to discriminate between benign and malignant lung lesions on CT scans. The main advantage of these parameters is that they have simple mathematical definitions and relatively easy/intuitive interpretation, as they can be seen as percentages of an overall form, respectively *elongated* (rod-like), *flat* (platy), and *compact* (equant). Furthermore, these parameters have all values in [0, 1], which facilitates clinical readings and comparisons.

We tested the above features along with three other form factors (Kong’s elongation and flatness and maximum projection sphericity), three conventional features (maximum 3D diameter, volume, and surface area), and two shape features (sphericity and volume density) for significant differences between benign and malignant lung nodules. Furthermore, we evaluated the effectiveness of these features within multivariate prediction models to discriminate between benign and malignant lesions.

The remainder of the paper is organised as follows. We describe the materials and methods in Section 2 including a description of the study population, the image acquisition and lesion delineation procedure, the feature extraction step, and the statistical analysis. We report the main results in Section 3 followed by a thorough discussion of the results in Section 4. We conclude the paper with some final considerations (Section 5), the main limitations of the study, and prospective future research (Section 6). Mathematical formulations of the imaging features are reported in Appendix A.

## 2. Materials and Methods

### 2.1. Study Population

We considered a total of 192 lung lesions from two retrospective datasets, denoted as ‘SSR-1’ and ‘LUNGx’ in this paper.

Dataset ‘SSR-1’ contained baseline CT scans of 109 lung nodules (38 benign, 71 malignant) from as many patients (45 females, 64 males, age = 68.3 ± 8.9 (44–84) year) who received a thoracic PET/CT at the Unit of Nuclear Medicine of the Università degli Studi di Sassari, Sassari, Italy, between November 2014 and May 2019. Benignity or malignancy was assessed via histological examination. The CT scans for attenuation correction were acquired in helicoidal mode on a Discovery 710 PET/CT system (GE Healthcare, Chicago, IL, USA) with the following settings: tube voltage 120 kVp, slice thickness 3.75 mm, spacing between slices 3.27 mm, in-plane inter-voxel spacing 1.37 mm in both directions, and image size 512 px × 512 px. Table 1 summarises the characteristics of the patient series; further details about the acquisition procedure are available in [51].

Dataset ‘LUNGx’ included 83 nodules (42 benign, 41 malignant) from 70 patients (42 females, 28 males, age = 60.2 ± 13.4 (18–84) year) who underwent thoracic CT examination at The University of Chicago, Chicago, IL, USA between February 2006 and May 2007. Benignity or malignancy was determined by followup imaging (stability over two years and/or spontaneous resolution were considered indicative of benignity) and/or histological assessment. The scans were obtained from different systems (see [62] for details), and the acquisition settings were: tube voltage 120–140 kVp, slice thickness 1.00 mm, spacing between slices 1.00 mm, in-plane inter-voxel spacing 0.55–0.90 mm in both directions, and image size 512 px × 512 px. This dataset is publicly accessible through The Cancer Imaging Archive (TCIA [63,64]). The characteristics of the patient series are reported in Table 2.

### 2.2. Lesion Delineation

**Figure 1:**
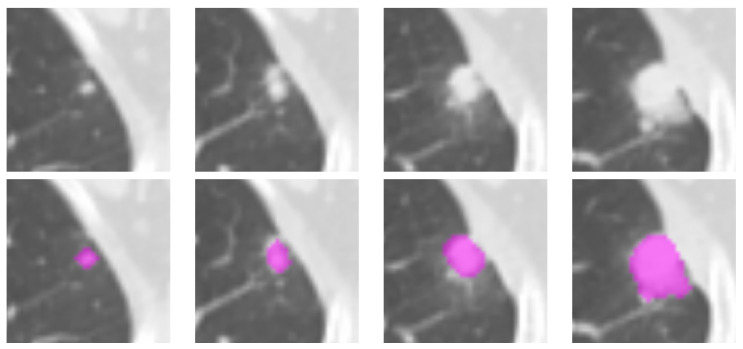
Illustration of the lesion delineation process. The top row shows the cropped areas from contiguous axial slices containing the suspicious lesion; the fuchsia overlays in the bottom row indicate the manually-delineated regions of interest. The lesion in the picture was diagnosed as adenocarcinoma in a 76-year-old man.

In both datasets the three-dimensional regions of interest (ROI) representing the suspicious areas were delineated manually, slice-by-slice, on the open-access LIFEx 7.1.0 platform [65], as shown in Figure 1. The segmentation was carried out together by two experts, one radiation oncologist (I.P., >15 year experience) and one nuclear medicine specialist (B.P., >20 year experience).

### 2.3. Shape Features

A total of 11 shape features were extracted from each ROI as detailed in Table 3. Mathematical definitions and formulae are reported in Appendix A. All the features, apart from the conventional ones, represented dimensionless quantities, and were, therefore, volume-independent by definition. Furthermore, they all had values in [0, 1], which facilitated empirical evaluations, comparisons, and potential translation into clinical practice.

**Figure 2:**
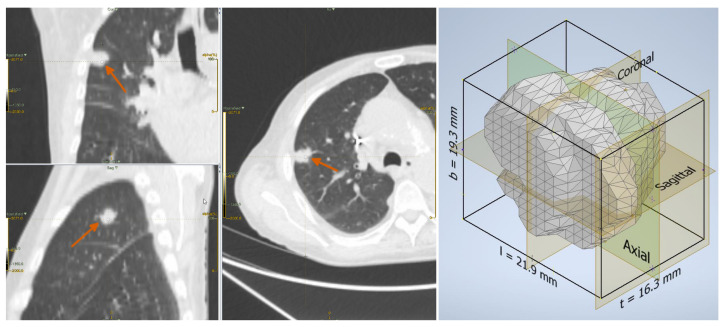
Adenocarcinoma in a 76-year-old man: lesion on the CT scan (**left**) and the reconstructed three-dimensional volume within the axis-aligned bounding box (**right**).

**Figure 3:**
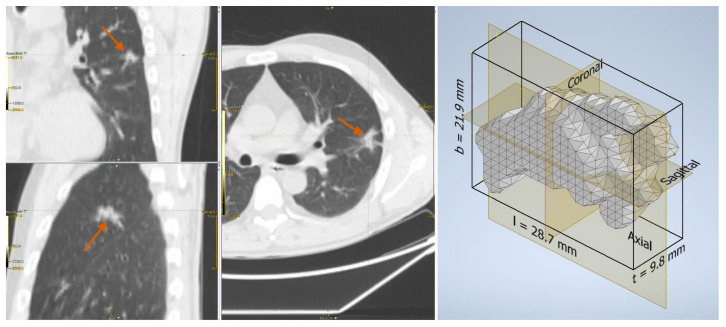
Fibrosis in a 46-year-old man: lesion on the CT scan (**left**) and the reconstructed three-dimensional volume within the axis-aligned bounding box (**right**).

As for the form factors, these were defined by the ratios of the three main dimensions of the lesion, which in the remainder we refer to as length (*l*), breadth (*b*), and thickness (*t*), with l≥b≥t. We took the side lengths (sorted in descending order of magnitude) of the rectangular axis-aligned bounding box of the ROI, respectively, as *l*, *b*, and *t* (also refer to Figure 2 and Figure 3 for a graphical explanation). Although this was a simplified way to compute these parameters (other approaches, for instance based on the principal axes of inertia, are also possible), it had the clear advantages of ease of calculation and straightforward interpretation.

### 2.4. Univariate Analysis

For each of the shape features described in Section 2.3, significant differences between the benign and malignant group were assessed by the nonparametric Mann–Whitney U test [66]. Correction for multiple tests was based on the Benjiamini–Hochberg procedure [67] at a false discovery ratio FDR=0.05.

### 2.5. Multivariate Prediction Models

The ability of the form factors to improve the discrimination capability between the benign and malignant lesions beyond standard imaging features was also assessed through multivariate prediction models. To this end, we considered two feature sets denoted as *base*, which included the conventional imaging features, that is, maximum 3D diameter, surface area, and volume, and *extended*, composed of all the features of the base set plus the form factors that were significantly different between the benign and malignant group in both datasets, which were: AFL, ACO, KFL, and MPS (see Table 4 and Table 5).

Prediction models based on linear Support Vector Machines (lSVM) were fitted and tested both internally (intra-dataset) and externally (across datasets) through four train/test combinations: SSR-1/SSR-1, LUNGx/LUNGx, SSR-1/LUNGx and LUNGx/SSR-1 (see Table 6 for the details of the results). Since the magnitude of the base features differed significantly from that of the form factors, all the features were preliminarily normalised to zero-mean and unit-variance (Z score). The normalisation was carried out feature by feature separately and independently on the two datasets (each dataset was blind to the data contained in the other one). The optimal value for the lSVM penalty factor *C* was determined through a grid search over C∈{0.01,0.1,1.0,10.0}. For each feature set + classifier combination, we retained the value of *C* that achieved the best performance. The performance of the prediction models was estimated as the percentage of nodules of the test set classified correctly (accuracy). For intra-dataset validation the split into train and test set was based on the leave-one-out procedure.

### 2.6. Estimation of the Cutoff Thresholds

To facilitate the interpretation of the form factors and further demonstrate their potential use on a practical level, we computed the optimal cutoff thresholds that maximised the overall classification accuracy over the two datasets considered separately and together. In Table 7, we provide the cutoff values for each of the form factors that were significantly different between the benign and malignant group.

## 3. Results

**Figure 4:**
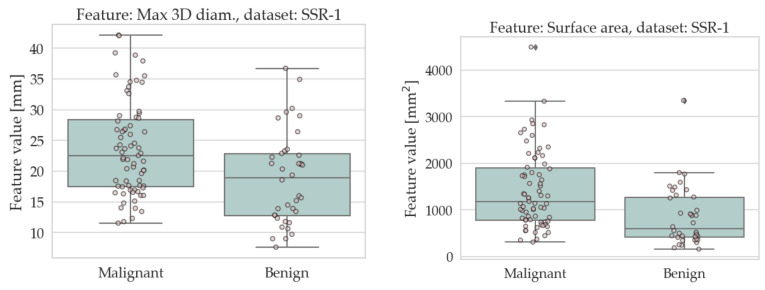

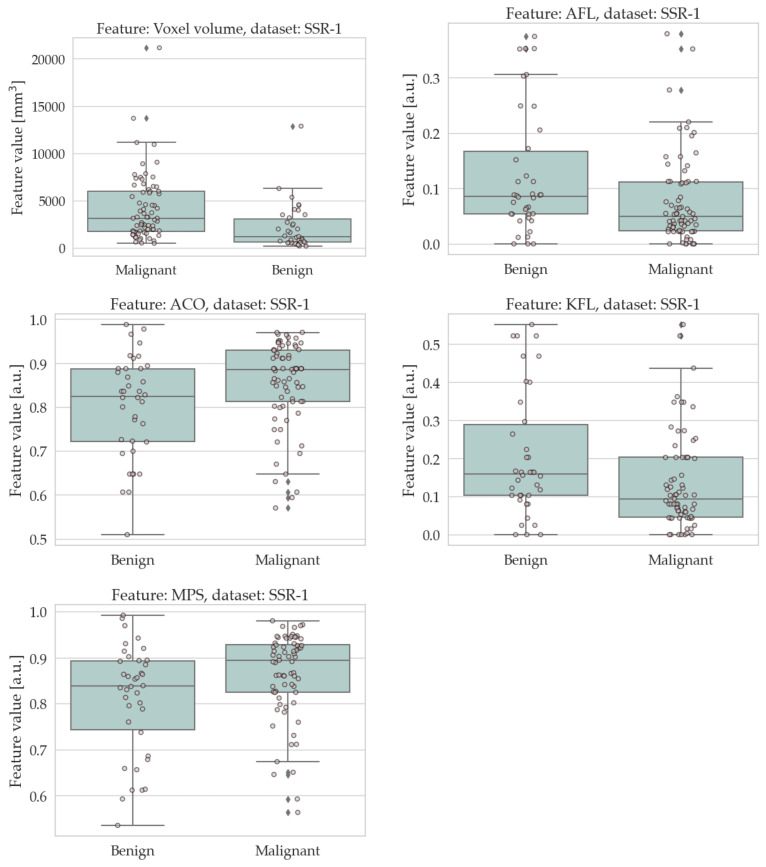
Boxplots/stripplots of the features that were significantly different between the benign and malignant tumours in the SSR-1 dataset.

**Figure 5:**
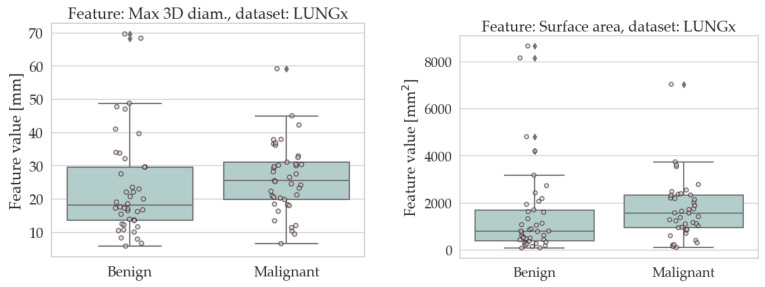

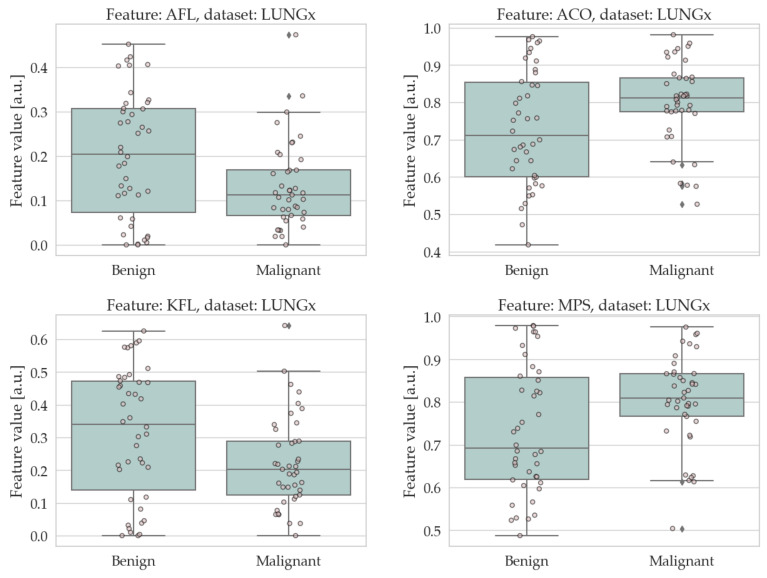
Boxplots/stripplots of the features that were significantly different between the benign and malignant tumours in the LUNGx dataset.

The results of the univariate analysis are summarised in Table 4 and Table 5; a visual representation of the data in the form of boxplots/stripplots is also available in Figure 4 and Figure 5. As can be seen, the malignant lesions were on average larger in both datasets, which is logical and consistent with the literature [40,68,69,70,71]. Regarding the form factors, four of them (AFL, ACO, KFL, and MPS) were significantly different between the two groups in both datasets. Specifically, AFL and KFL were higher in the benign group, whereas ACO and MPS were higher in the malignant group. In other words, the benign lesions were, on average, flatter than the malignant ones; conversely, the malignant ones were more isotropic (equant) than the benign ones. The other two shape features considered in this study (sphericity and volume density) did not show statistically significant differences between the two groups in either dataset.

Table 6 shows the accuracy of the multivariate prediction models built upon the *base* and *extended* feature sets as described in Section 2.5. We would like to emphasize that it is not the absolute accuracy value that matters here (ideally, this could be increased by adding more clinical and/or radiomics features) but the gain that could be obtained by adding the form factors to the base features. This ranged between 0.9 pp and 14.5 pp and was particularly pronounced when the LUNGx dataset was used as a training set. This is interesting, as this dataset was specifically designed for a competition (‘LUNGx Challenge for Computerized Lung Nodule Classification’) and is considered particularly difficult [62,72].

## 4. Discussion

In recent years the use of quantitative imaging features coupled with automatic classifiers has gained considerable attention as a means to assist the clinician in the diagnosis and management of suspicious lung lesions. In this context, shape descriptors have been investigated as potential imaging biomarkers to differentiate benign vs. malignant lung lesions on CT scans, since morphological features such as irregular borders and spiculation are known to be associated with malignancy [6,7,73]. Consequently, most previous studies have focused on how to quantify these features by suitable mathematical parameters [44,46,48].

Our results suggest a potential link between overall lesion shape and benignity/malignancy. Specifically, we found that lesion flatness was associated with benignity and compactness (equancy) with malignancy. This is congruent with the findings reported by Takashima et al. [74], where the manually-assessed three-dimensional shape ratio was significantly different between benign and malignant lesions, with the latter again leaning towards equancy. Our result for flatness also confirmed the one reported by Peikert et al. [75], although the authors did not discuss this finding further, as their work focused on a multiparametric classification model and not on the individual features. Regarding the KEL and KFL, a comparison with the literature indicates that our findings were again in good agreement with those presented by Peikert et al. [75]. No comparison was possible for the other form factors of AEL, ACO, AFL, and MPS, as we are not aware of any previous study investigating these parameters. Finally, sphericity and volume density were not statistically significant in our study. The result for sphericity contrasted with Dhara et al. [55], where this parameter was significantly different between the benign and malignant group, although in [55] it is not indicated which group had the higher values.

From a clinical standpoint, the most relevant finding of this work is that the benign lesions had on average a tendency to be flatter than the malignant ones; conversely, the malignant ones leaned toward a more isotropic (equant) morphology. We demonstrated that four form factors among those investigated here were significantly different between the benign and malignant group in both datasets, suggesting that they could be used in clinical decision making. To clarify the potential use of these parameters, we have reported the optimal cutoff values for benignity/malignancy for each of the form factors that were significantly different between the benign and malignant lesions. However, further studies, ideally prospective and on larger cohorts of patients, are needed to confirm these findings before translation into clinical practice.

## 5. Conclusions

The diagnostic evaluation of suspicious lung nodules detected on CT scans represents a significant challenge for the clinician. The traditional radiographic approach involves manual assessment of specific features such as size, contour, margins, internal characteristics, spiculation, and lobulation. In recent years, the quantitative analysis of imaging data coupled with machine learning algorithms (radiomics) has opened up new perspectives in the field. In this scenario, the objective of this work was to investigate one specific subset of morphological features (form factors) as potential imaging biomarkers to discriminate between benign and malignant lung lesions on CT scans.

We found that four form factors (ACO, AFL, KFL, and MPS) were significantly different between the benign and malignant groups in both datasets. Furthermore, we demonstrated that these parameters could improve the accuracy of automated classification models for discriminating benign vs. malignant lesions. Our findings lead to the speculation that malignant lesions have a tendency to grow more isotropically than the benign ones. This hypothesis, however, needs to be validated in future studies. Future work should also address potential links between tumour microenvironment and overall shape.

## 6. Limitations and Future Work

This work was not exempt from limitations; two of the limitations were the retrospective nature and the relatively contained sample size. The results should be validated in larger and, ideally, prospective studies. The biological links between overall shape as quantified by the form factors (particularly in terms of flatness vs. equancy) and the potentially different spatial growth patterns for malignant and benign lesions also remain unclear and should be investigated in future studies.

## Figures and Tables

**Table 1:** Dataset SSR-1: Characteristics of the patient series.

Attribute [Data Format]	Value
*Demographics*	
Age [Mean ± SD]	68.3 ± 8.9 year
Female [*N* (%)]	45 (41.3)
Male [*N* (%)]	64 (58.7)
*Histology*	
Benign [*N* (%)]	38 (34.9)
Malignant [*N* (%)]	71 (65.1)
Adenocarcinoma [*N* (%)]	45 (41.3)
Atypical carcinoid (NSCLC) [*N* (%)]	1 (0.9)
Metastasis [*N* (%)]	1 (0.9)
Neuroendocrine tumour [*N* (%)]	1 (0.9)
Small-cell lung cancer [*N* (%)]	2 (1.8)
Spinocellular carcinoma [*N* (%)]	4 (3.7)
Squamous cell carcinoma [*N* (%)]	9 (8.3)
Unspecified [*N* (%)]	8 (7.3)

**Table 2:** Dataset LUNGx: Characteristics of the patient series.

Attribute [Data Format]	Value
*Demographics*	
Age [Mean ± SD]	60.2 ± 13.4 year
Female [*N* (%)]	42
Male [*N* (%)]	28
*Histology*	
Benign [*N* (%)]	42 (50.6)
Malignant [*N* (%)]	41 (49.4)
Adenocarcinoma [*N* (%)]	17 (20.5)
Carcinoid tumour [*N* (%)]	2 (2.4)
Small-cell lung cancer [*N* (%)]	9 (10.8)
Squamous cell carcinoma [*N* (%)]	1 (1.2)
Suspicious lung cancer [*N* (%)]	2 (2.4)
Unspecified NSCLC [*N* (%)]	10 (12.0)

**Table 3:** Summary table of the shape features considered in this study (see Appendix A for the mathematical definitions and formulae).

Group	Name	Acronym/Abbreviation
Conventional	Maximum 3D diameter	Max3Ddiam
	Surface area	SurfArea
	Voxel volume	Volume
Form factors	Angelidakis elongation	AEL
	Angelidakis flatness	AFL
	Angelidakis compactness	ACO
	Kong elongation	KEL
	Kong flatness	KFL
	Maximum projection sphericity	MPS
Other	Sphericity	-
	Volume density	VDN

**Table 4:** Results of the univariate analysis on the SSR-1 dataset. Units of measure: maximum 3D diameter [mm], surface area [mm${}^{2}$], and volume [mm${}^{3}$]; all other features are in dimensionless units (range 0–1).

Feature	Benign	Malignant	*p*-Value	Significant
Max 3D diameter	18.8 ± 7.4	23.6 ± 7.7	0.001	Yes
Surface area	846.7 ± 630.3	1414.4 ± 819.6	<0.001	Yes
Voxel volume	2138.1 ± 2369.2	4209.2 ± 3481.3	<0.001	Yes
Angelidakis elongation	0.077 ± 0.056	0.070 ± 0.059	0.193	No
Angelidakis flatness	0.123 ± 0.111	0.077 ± 0.079	0.009	Yes
Angelidakis compactness	0.800 ± 0.115	0.853 ± 0.097	0.008	Yes
Kong elongation	0.140 ± 0.096	0.126 ± 0.099	0.200	No
Kong flatness	0.205 ± 0.163	0.136 ± 0.124	0.010	Yes
Maximum projection sphericity	0.810 ± 0.117	0.864 ± 0.092	0.005	Yes
Sphericity	0.774 ± 0.067	0.769 ± 0.061	0.280	No
Volume density	0.435 ± 0.112	0.431 ± 0.097	0.274	No

**Table 5:** Results of the univariate analysis on the LUNGx dataset. Units of measure: maximum 3D diameter [mm], surface area [mm${}^{2}$], and volume [mm${}^{3}$]; all other features are in dimensionless units (range 0–1).

Feature	Benign	Malignant	*p*-Value	Significant
Max 3D diameter	23.5 ± 15.1	26.1 ± 10.4	0.029	No
Surface area	1457.2 ± 1882.1	1698.9 ± 1252.6	0.012	Yes
Voxel volume	2782.5 ± 4550.9	3436.0 ± 3432.3	0.011	Yes
Angelidakis elongation	0.070 ± 0.078	0.069 ± 0.059	0.334	No
Angelidakis flatness	0.201 ± 0.139	0.132 ± 0.096	0.015	Yes
Angelidakis compactness	0.730 ± 0.152	0.799 ± 0.110	0.017	Yes
Kong elongation	0.127 ± 0.126	0.126 ± 0.103	0.382	No
Kong flatness	0.315 ± 0.198	0.224 ± 0.139	0.014	Yes
Maximum projection sphericity	0.734 ± 0.148	0.803 ± 0.105	0.019	Yes
Sphericity	0.662 ± 0.129	0.625 ± 0.087	0.036	No
Volume density	0.359 ± 0.096	0.339 ± 0.071	0.047	No

**Table 6:** Performance of the classification models. Accuracy columns report the percentage (fraction) of the samples of the test set classified correctly; the gain is the difference between the base and extended feature sets.

Training Set	Test Set	Accuracy (*Base*) [% (Fraction)]	Accuracy (*Extended*) [% (Fraction)]	Gain [pp (Fraction)]
SSR-1	SSR-1	65.1 (71/109)	66.1 (72/109)	0.9 (1/109)
LUNGx	LUNGx	54.2 (45/83)	62.7 (52/83)	8.4 (7/83)
SSR-1	LUNGx	49.4 (41/83)	63.8 (53/83)	14.5 (12/83)
LUNGx	SSR-1	57.8 (63/109)	63.9 (71/109)	7.3 (8/109)

**Table 7:** Estimated cutoff values for malignancy. The range for all parameters is 0–1.

Feature	Dataset			Avg. over Datasets
	SSR-1	LUNGx	SSR-1 + LUNGx	
ACO	>0.746	>0.765	>0.769	>0.760
AFL	<0.245	<0.248	<0.248	<0.246
KFL	<0.368	<0.415	<0.396	<0.393
MPS	>0.697	>0.701	>0.706	>0.701

## Data Availability

The data presented in this study are available on request from the corresponding author.

## Abbreviations

The following abbreviations are used in this manuscript:

ACO	Angelidakis compactness
AEL	Angelidakis elongation
AFL	Angelidakis flatness
CT	Computed Tomography
FDR	False discovery ratio
KEL	Kong elongation
KFL	Kong flatness
MPS	Maximum projection sphericity
NSCLC	Non-Small Cell Lung Cancer
PET	Positron Emission Tomography
ROI	Region(s) of interest
SCLC	Small Cell Lung Cancer
lSVM	Linear Support Vector Machines
TCIA	The Cancer Imaging Archive

## Appendix A. Shape Features

### Appendix A.1. Conventional

#### Appendix A.1.1. Voxel Volume

The total volume defined as the sum of the volume of each voxel in the region of interest. This is indicated as *V* in the remainder of this Appendix A.

#### Appendix A.1.2. Surface Area

The total area of the triangular mesh that approximates the boundary of the region of interest. This is indicated as *A* in the remainder of this Appendix A.

#### Appendix A.1.3. Maximum 3D Diameter

The Euclidean distance between the centroids of the two most apart voxels in the region of interest.

### Appendix A.2. Form Factors

Let *l*, *b*, and *t* denote the side lengths of the axis-aligned bounding box enclosing the region of interest sorted in descending order (l≥b≥t).

Angelidakis compactness, elongation, and flatness (ACO, AEL, and AFL)

(A1) $$\begin{array}{ccc} \mathrm{ACO} & = & \frac{2t}{l+t} \end{array}$$

(A2) $$\begin{array}{ccc} \mathrm{AEL} & = & \frac{lt}{lt+b^{2}}-\frac{t}{l+t} \end{array}$$

(A3) $$\begin{array}{ccc} \mathrm{AFL} & = & \frac{b^{2}}{lt+b^{2}}-\frac{t}{l+t} \end{array}$$

By definition AEL, AFL, and ACO have all values in (0, 1] and add up to unity [61]. Larger values of AEL, AFL, and ACO, respectively, indicate higher elongation, flatness, and compactness. The main advantage of these parameters is that they can be seen as percentages of an overall form, respectively, rod-like, platy, and equant.

Kong elongation and flatness

(A4) $$\begin{array}{ccc} \mathrm{KEL} & = & 1-\frac{b}{l} \end{array}$$

(A5) $$\begin{array}{ccc} \mathrm{KFL} & = & 1-\frac{t}{b} \end{array}$$

Kong elongation and flatness [76] are a variation of the classic breadth-to-length and thickness-to-breadth form factors [77]. The advantage of KEL and KFL is that a flat particle has a high value of KEL, and an elongated one has a high value of KFL.

Maximum projection sphericity

(A6) $$\begin{array}{ccc} \mathrm{MPS} & = & \sqrt[{3}]{\frac{t^{2}}{bl}} \end{array}$$

The ratio between the maximum projection area of the region of interest and that of a sphere with the same volume. It reflects the difference of forces (drag and gravitational) of a body immersed in a fluid [78]. It is a measure of equancy.

### Appendix A.3. Others

Sphericity

(A7) $$\frac{\sqrt[{3}]{36\pi V^{2}}}{A}$$

The ratio between the surface area of a sphere with the same volume as the region of interest and the surface area of the region of interest.

Volume density

(A8) $$\frac{V}{V_{aabb}}$$

The ratio between the volume of the region of interest and that of the axis-aligned bounding box ($V_{aabb}$).
